# ePro-ClearSee: a simple immunohistochemical method that does not require sectioning of plant samples

**DOI:** 10.1038/srep42203

**Published:** 2017-02-08

**Authors:** Kiyotaka Nagaki, Naoki Yamaji, Minoru Murata

**Affiliations:** 1 Institute of Plant Science and Resources, Okayama University, Kurashiki 710-0046, Japan

## Abstract

Investigations into the epigenetic status of individual cells within tissues can produce both epigenetic data for different cell types and positional information of the cells. Thus, these investigations are important for understanding the intra- and inter-cellular control systems of developmental and environmental responses in plants. However, a simple method to detect epigenetic modifications of individual cells in plant tissues is not yet available because detection of the modifications requires immunohistochemistry using specific antibodies. In this study, we developed a simple immunohistochemical method that does not require sectioning to investigate epigenetic modifications. This method uses a clearing system to detect methylated histones, acetylated histones, methylated DNA and/or centromeric histone H3 variants. Analyses of four dicots and five monocots indicated that this method provides a universal technique to investigate epigenetic modifications in diverse plant species.

Loci on individual chromosomes are occasionally controlled differentially by different epigenetic modifications depending on the environmental conditions and cell positions, even in the same organism. To elucidate these phenomena, the understandings of not only the epigenetic modifications in each cell but also of the spatio-temporal relationships of the cells are necessary.

Epigenetic modifications have been analyzed primarily by chromatin immunoprecipitation (ChIP) using antibodies raised against specific epigenetic modifications^1^,^2^,^3^,^4^,^5^. However, ChIP analyses are unable to elucidate the epigenetic modifications in individual cells because the analyses are performed for a mixture of cells, and the results appear as averages of the modification status on chromosomal loci among the cells analyzed.

Recently, epigenetic modifications in individual cells were investigated by immunohistochemical staining using sliced plant tissues^2^. However, the antibodies penetrated only into cells on the surface of the slices, and the detection of epigenetic status could be investigated only in the cells that the antibodies penetrated. In addition, immunohistochemical staining is not applicable for tissues that are difficult to slice, e.g., cross-sections of leaves.

Clearing is a method used to make tissues transparent to investigate cells *in situ*, and it has been used for more than 100 years since it was first attempted^6^. However, reagents used in classical clearing methods are harmful to fluorescent proteins and antigen proteins. Recently, a number of clearing methods that are not harmful to fluorescent proteins have been developed to investigate the neural networks of animal brains^7^,^8^,^9^,^10^. However, these clearing methods are not easy to apply directly to plant tissues given the existence of chloroplasts and cell walls, both of which emit strong autofluorescence^11^. In addition, rigid cell walls also obstruct antibody penetration into the cells^12^.

To overcome the autofluorescence problems, a couple of clearing methods for plant tissues have been developed recently^12^,^13^,^14^. Among these methods, the ClearSee method and TOMEI (Transparent plant organ method for imaging) remove colors, including those from chlorophylls, and reduce light scattering using reagents that have high refractive indexes^13^,^14^. These methods make fluorescent proteins visible in leaves, roots, pistils and flowers. However, the methods have never been applied for immunohistochemical staining that is essential for investigating epigenetic modifications because the modifications are undetectable using fluorescent proteins.

Another method, PEA-CLARITY, which was developed from the CLARITY method for animal tissues, has solved problems relating to autofluorescence and antibody accessibility^7^,^12^. However, this method requires numerous preparation steps: embedding in poly-acrylamide gels, cross-linking to the poly-acrylamide gels, removing lipids and colors using a reagent containing SDS (sodium dodecyl sulfate), and digesting cell walls and starches with enzymes to create spaces for antibody access. Thus, a considerable amount of time is required (7–9 weeks) to complete the processes. In addition, only RuBisCo (Ribulose 1,5-bisphosphate carboxylase/oxygenase), one of the most abundant proteins in plant leaves^15^, has been detected by this method^12^. Therefor, whether the method has enough power to detect epigenetic modifications remains uncertain because immunosignals of the modifications are weaker than those of RuBisCo. Another immunocytochemical method for plant whole-mount samples^16^ needs a shorter time (2–3 days) than the PEA-CLARITY, but consists a number of steps with frequent changes of solutions, including toxic reagents (methanol, xylol and dimethylsulfoxide). This method has never been applied to detect epigenetic modifications.

In this study, we developed a new clearing method for immunohistochemistry using enzymes and 2-Propanol treatments before ClearSee clearing (ePro-ClearSee). Using this method without harmful reagents, modified histones and DNA and centromere-specific histone variants were detected clearly in leaves and roots without sectioning for duration of 10 days to 3 weeks.

## Results

### Clearing

**Figure 1:**
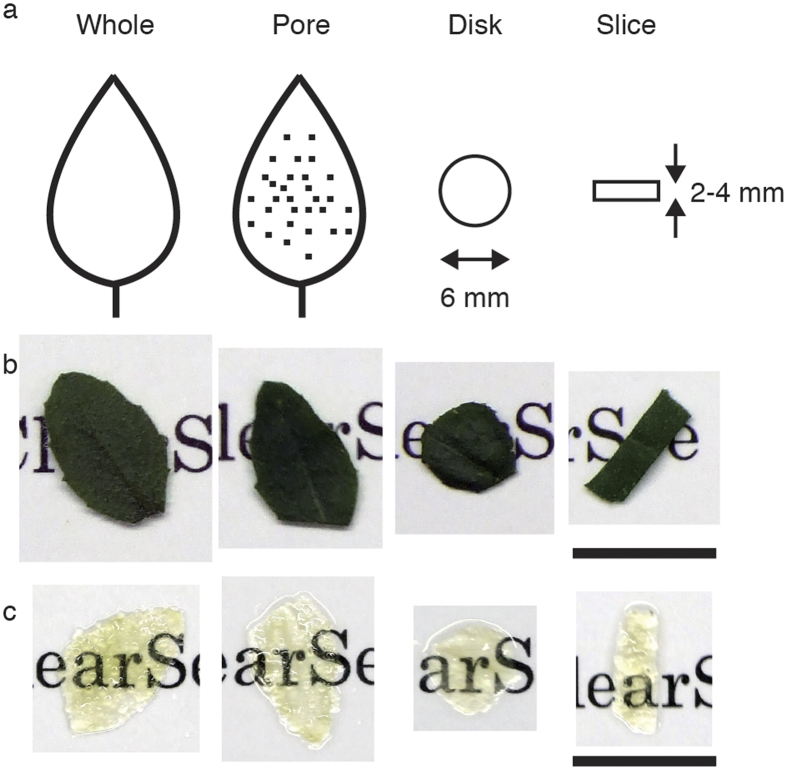
Clearing of *Arabidopsis* leaves by the ePro-ClearSee method. Four different shapes (**a**) of leaves were cleared by the ePro-ClearSee method. Leaves before (**b**) and after (**c**) clearing are indicated. Scale bar, 1 cm.

The ePro-ClearSee method was applied to four dicot and five monocot species (Table 1). Leaves and roots were used in this experiment, and leaves with the following four shapes were treated: whole, pore, disk and slice (Fig. 1a and b, and Figure S1). ‘Pore’ indicates that the leaves were perforated with a tip of a disposable injection needle. ‘Disk’ indicates that 6-mm diameter leaf disks were punched out by a hole-puncher. In the case of ‘slice’, leaves were cut 2 to 4 mm in width. Digestion with enzymes and clearing with 2-propanol and ClearSee made the leaves transparent (Fig. 1c). The ClearSee treatment times required for sufficient transparency varied (1–7 days), depending on the shapes and species (Table 1). Perforating reduced the clearing time in all six tested species (Table 1).

### Immunohistochemistry

**Figure 2:**
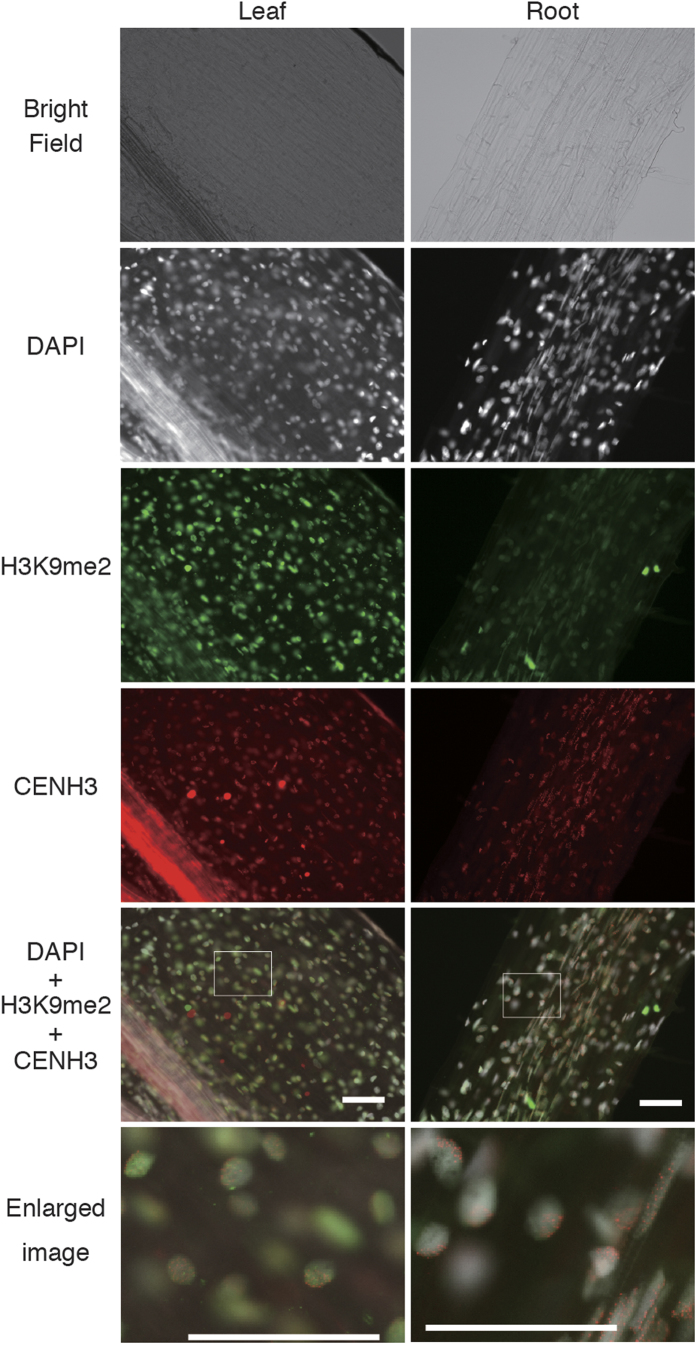
Two-dimensional imaging of an ePro-ClearSee treated wheat leaf and root depicting immunosignals. Immunosignals of di-methylated histone H3 at Lys9 (green) and CENH3 (red) were visualized with DAPI stained nuclei (gray). Scale bar, 100 μm.

After clearing, the tissues were washed and subjected to immunohistochemistry. First, potential of ClearSee and alcohols for clearing and penetration were tested using anti-centromere-specific histone H3 variants (CENH3) and anti-histone H3 dimethylated at lysine 9 (H3K9me2) antibodies to wheat leaves (Figure S2). The ClearSee treatment reduced autofluorescence, but penetration of the antibodies was not enough. To increase accessibility of the antibodies, a permeabilization step with methanol or 2-propanol was added between cell wall digestion and ClearSee treatments. Methanol treatment reduced red autofluorescence, but did not produce enough accessibility of the antibodies. On the other hand, 2-propanol increased accessibility of the antibodies. Although similar levels of the accessibility could be obtained only by a 2-propanpl treatment without ClearSee, strong autofluorescence was left. Therefore, the sequential treatment with enzyme, 2-propanol and ClearSee (ePro-ClearSee) steps were selected, and applied to detect these two proteins and other antigens including tubulin, modified histones [histone H3 dimethylated at lysine 4 (H3K4me2) and acetylated histone H4 (H4Ac)] and 5-methylcytidine (5meC) in the species. For all antibodies and species used, immunosignals detectability was summarized in Table 2. Although there are many different reasons for undetected immunosignals (e.g. low abundance or denature of antigen, insufficient tissue permeabilization and low affinity of antibody)^16^, these can estimate from the results. For all of the antibodies used, signals could be detected in at least one species (Table 2 and Fig. 2, S2–S8), suggesting that the clearing method did not affect the antigens. Since microtubule stabilization buffer was used in a fixative of this method, nuclei were fixed to cytoskeleton consisting of tubulin (Figure S3, S5 and S6). In some whole leaves and disks, the signals were detected only around the cut edges, wounded regions and vascular bundles (Figure S3, S5–S8), suggesting that the antibodies applied could enter mainly from mechanically damaged regions via vascular bundles. Similarly, the perforating step is thought to increase the antibody penetration (Figure S8). Although only local positional information of cells is retained in the ‘slice’, the most intense immunosignals were observed in this shape. Based on these results, it is better to select shapes depending on the purpose of the study. ‘Slice’ is the better shape for clear and less-damaged imaging in small parts of tissues, and ‘pore’ is a better shape to obtain a bird’s-eye view of the epigenetic information in a leaf.

### Advantages of ePro-ClearSee

The ePro-ClearSee established in this study is of great advantage to the PEA-CLARITY in treatment time. To know more advantages of the ePro-ClearSee, the fastest immunohistochemical method reported by Sauer *et al*.^16^ was compared using barley leaves (Figure S3 and S10). The samples prepared by Sauer’s method showed much stronger green and blue autofluorescence than those by ePro-ClearSee, though the red autofluorescence was a little weaker. One of the main advantages ePro-ClearSee was good antibody accessibility that was poor in Sauer’s method.

### Fluorescent protein detection

Detectability of fluorescent protein was tested using a transgenic tomato expressing green fluorescent protein fused to CENH3 (GFP::CENH3). Dehydration is known to eliminate fluorescence of fluorescent proteins, the fluorescence did not remain after the 2-propanol treatment (Figure S7). However, the GFP::CENH3 was detectable by combination with anti-GFP antibody (Figure S7).

### Transparency assessment

**Figure 3:**
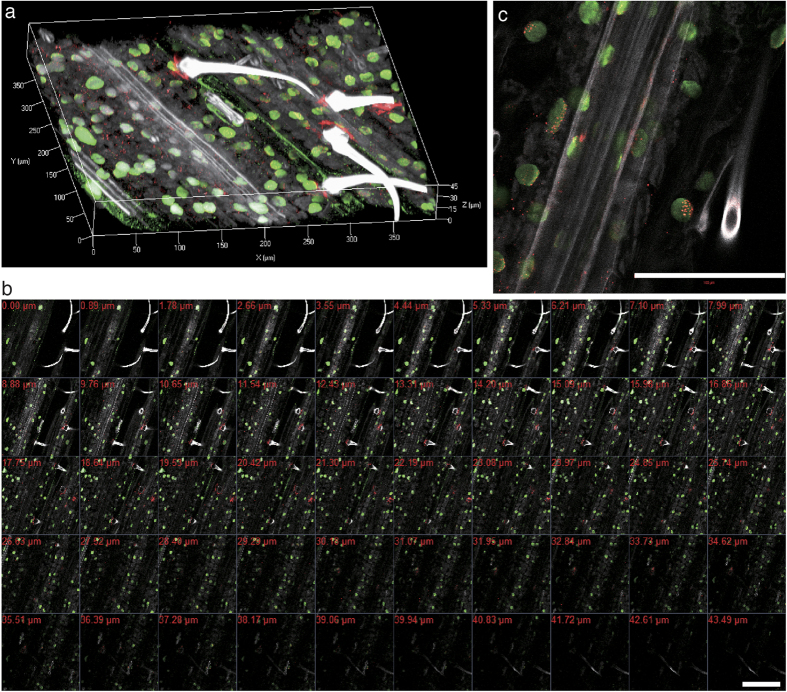
Three-dimensional imaging of an ePro-ClearSee treated wheat leaf depicting immunosignals. Immunosignals of di-methylated histone H3 at Lys9 (green) and CENH3 (red) were visualized with DAPI stained nuclei (gray). A three-dimensional projection (**a**) of a wheat leaf was constructed from optical CSLM sections (**b**). A close-up image of the optical section (**c**). Scale bar, 100 μm.

To assess transparency of the cleared tissues, leaves and roots were scanned by confocal laser scanning microscopy (CLSM). In wheat, both signals from H3K9me2 and CENH3 were detected throughout the entire leaf thickness (<40 μm) (Fig. 3 and Movie S1). A similar transparency (approximately 50 μm in depth) was observed in garlic leaves (Figure S10a). For garlic roots, H3K9me2 signals were detectable even in a 200 μm depth area (Figure S10b).

### High-resolution analyses

**Figure 4:**
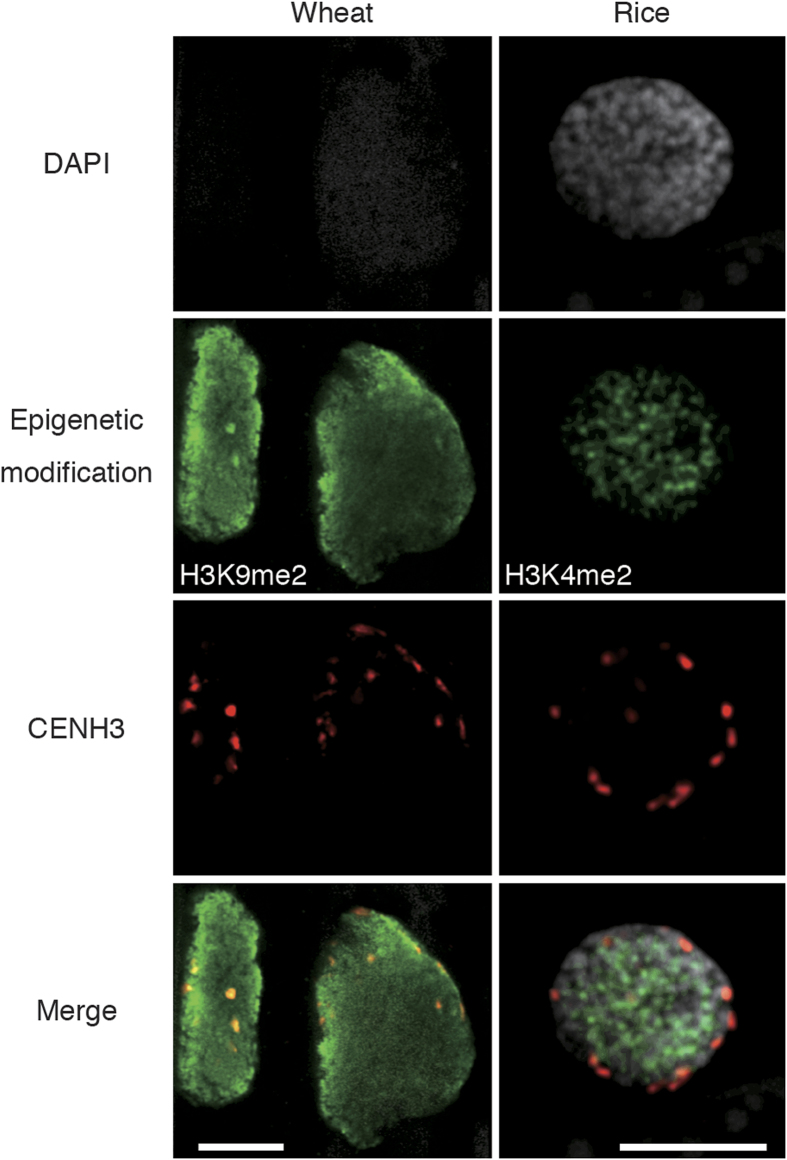
High-resolution imaging of ePro-ClearSee treated wheat and rice leaves depicting immunosignals. Immunosignals of di-methylated histone H3 at Lys9 or Lys4 (green) and CENH3 (red) were visualized with DAPI stained nuclei (gray). Scale bar, 10 μm.

High-resolution analyses by CLSM typically require more intensive signals than those in standard CLSM analyses. The suitability of samples cleared by the ePro-ClearSee method for high-resolution analyses were tested using LSM800 with Airyscan; cleared wheat and rice leaves were analyzed using this method (Fig. 4). In wheat, CENH3 signals and intensive H3K9me2 signals were observed around the nuclear membrane. Also, the similar centromere positioning of the wheat result and intensive H3K4me2 signals in the internal area of nuclei were observed in rice. These results suggest that the clearing method is suitable for high-resolution analyses by CLSM.

## Discussion

In this study, we developed a clearing method for immunohistochemical (ePro-ClearSee) analyses. The ePro-ClearSee method enabled the detection of immunosignals 200 μm deep in plant tissues without the need for sectioning for a short period of time (10 days to 3 weeks) (Table 3 and Figure S9). For immunohistochemical analyses, the best result was obtained by PEA-CLARITY, but it took 7–9 weeks^12^. In addition, almost all processes were conducted at 37 °C (Table 3). The long period and high temperature increase the risk of degradation of target proteins. By contrast, the microtubule stabilization buffer fixative in the ePro-ClearSee method could fix nuclei to the cytoskeleton (Figure S3, S5 and S6). This superiority made it possible for the ePro-ClearSee method to omit a hydrogel polymerization step that is essential in PEA-CLARITY, and the gel-less fixation allowed for the more rapid penetration of enzymes and antibodies into the tissues. Hence, ePro-ClearSee reduced the time required to detect immunosignals by approximately one-third to one-fifth compared with PEA-CLARITY (Table 3). In addition, treatments at 37 °C in ePro-ClearSee method were only 3 h. These changes are helpful in detecting unstable and/or minor targets. As the results, epigenetic modifications, including H3K4me2, H3K9me2, H4Ac and 5meC, were clearly detected in this study by introducing an improved clearing method (Fig. 2, S2–S8). Although the fastest immunohistochemical method reported by Sauer *et al*. needs only 2–3 days to obtain immunosignals^16^, the detection ability is not enough to analyze the epigenetic modifications (Figure S9). In addition, ePro-ClearSee method worked in a wide range of plant species (Fig. 2, S2–S8), suggesting that the method may be applicable to detect epigenetic modifications in all plant species.

In addition to epigenetic analyses, the ePro-ClearSee method is also applicable to other analyses. For example, CENH3 signals and volume information of the nuclei are useful to estimate ploidy of individual cells in tissues, and the features are useful to investigate polyploid cells in different tissues. We did not apply this method to detect proteins on cell membrane or in organelles, if the method applicable to detect these proteins, but it is highly possible to detect them because this method uses quite mild treatments to clean compared with the other methods.

## Materials and Methods

### Plant Material

Wheat (*Triticum aestivum* cv. Chinese Spring), barley (*Hordeum vulgare* cv. Betzes), maize (*Zea mays* cv. GoldRush) and rice (*Oryza sativa* cv. Nipponbare) were germinated and grown for 1 week on filter paper. Garlic (*Allium sativum*) was germinated and grown by hydroponic culture for one week. *Arabidopsis thaliana* ecotype Col-0, tobacco (*Nicotiana tabacum*), tomato (*Solanum lycopersicum* cv. Micro-Tom) and sunflower (*Helianthus annuus)* were germinated and grown in pots with soil for one month. Tomato seeds (TOMJPF00001) were provided by University of Tsukuba Gene Research Center through the National Bio-Resource Project of the MEXT, Japan. Leaves were collected from the 1-week-old seedlings or the 1-month-old plants. Wheat and garlic roots were collected from the seedlings.

Tomato plants expressing GFP-fused CENH3 were produced by an *Agrobacterium*-mediated transformation using Micro-Tom^17^. First, a PCR-amplified SlCENH3 cDNA (GenBank number: XM_010328624) was cloned into a binary vector pK7WGF2^18^ using Gateway system (Thermo Fisher Scientific, MA, USA), and then a generated clone was used for the plant transformation with *Agrobacterium tumefaciens*. Leaves were collected from a one-month-old transformant.

### Fixation

Collected leaves and roots were soaked in a fixative (microtubule-stabilizing buffer (50 mM PIPES, pH 6.9, 5 mM MgSO_4_, and 5 mM EGTA) containing 3% (w/v) paraformaldehyde and 0.3% (v/v) Triton X-100). Penetration of the fixative into the tissues was achieved by subjecting the samples to three cycles of vacuum (−50 kPa) for 2 min each with release. Then, the plant tissues were fixed in the fixative for 15 min at room temperature. After fixation, the tissues were washed twice in PBS for 10 min at 4 °C. In the ‘pore’, the leaves were perforated using a tip of a disposable 24-gauge needle.

### Digestion

The fixed tissues were soaked in 1% (w/v) cellulase Onozuka RS (Yakult Pharmaceutical Industry, Tokyo, Japan) and 0.5% (w/v) pectolyase Y-23 (Seishin Pharmaceuticals, Tokyo, Japan) mixture dissolved in PBS with 0.3% (v/v) Triton X-100. Penetration of the enzyme mixture into the tissues was achieved by subjecting the samples to five cycles of vacuum (−50 kPa) for 2 min each with release. Then, the tissues were incubated for 30 to 60 min at 37 °C and washed twice in PBS for 10 min each at 4 °C.

### Clearing

The digested tissues were washed in 2-propanol for 1 h at room temperature and then cleared by ClearSee solution [10% (w/v) xylitol, 15% (w/v) sodium deoxycholate and 25% (w/v) urea in water] at room temperature for 1 to 7 days. The ClearSee solution was changed once daily. After the tissues were cleared, the tissues were rinsed with 50% (v/v) 2-propanol in water for 10 min at room temperature and were washed twice in PBS for 10 min at 4 °C. To evaluate detectability, Sauer’s method was conducted using barley leaves as described in the protocol^16^.

### Immuno-localization

In primary antibody solutions, the following antibodies were diluted to 1:100 with a blocking solution [100 mM Tris-HCl, 150 mM NaCl and 0.5% (w/v) blocking reagent (Sigma-Aldrich, St. Louis, MO, USA)]: anti-α-tubulin mouse antibody (Sigma-Aldrich: T6199), anti-H3K9me2 mouse antibody (Abcam, Cambridge, UK: ab1220), anti-H3K4me2 mouse antibody (MBL, Nagoya, Japan: MABI0303), anti-H4Ac rabbit antibody (Millipore, Billerica, MA, USA: #06–598), anti-5meC mouse antibody (BIO-RAD, Oxford, UK: MCA2201), a anti-GFP mouse antibody (Acris, CA, USA: R1461P) for the GFP fused CENH3 in tomato, anti-OsCENH3 rabbit antibody^1^ for CENH3s in wheat, barley and rice, and anti-HaCENH3 rabbit antibody^2^ for CENH3 in sunflower. The cleared tissues were soaked in the primary antibody solutions, and penetration of the antibody mixture into the tissues was achieved by subjecting the samples to five cycles of vacuum (−50 kPa) for 2 min each with release. Then, the tissues were incubated for 3 to 7 days at 4 °C and 1 h at 37 °C and washed twice in PBS for 4 h at 4 °C. The secondary antibodies used were Alexa Fluor 555-labeled anti-rabbit antibodies (Molecular Probes, Eugene, OR, USA) and Alexa Fluor 488-labeled anti-mouse antibodies (Molecular Probes). Both antibodies were diluted to 1:500 with the blocking solution, penetrated cells, reacted and washed the same procedure as the primary antibodies. The tissues were transferred onto slide glass and mounted by an anti-fade, SlowFade Diamond containing 1 μg/ml DAPI (Thermo Fisher Scientific). The slides were placed at 4 °C for least one day, until the anti-fading solution penetrated completely into the tissues. Immunosignals were observed with a fluorescence microscope (Keyence, Osaka, Japan; BZ-9000) using Nikon CFI Plan Apochromat 20x and 40x objectives for two-dimensional analyses and CLSM (Carl Zeiss, Jena, Germany; LSM700 and LSM800 with Airyscan) using Plan-Apochromat 20x, LD LC2 Plan-Apochromat 25x and Plan-Apochromat 63x for three-dimensional and high-resolution analyses analyses. In the two-dimensional analyses, images were captured using ‘Multi-colour image capturing software’ build in the BZ-9000 system, and the captured images were overlaid using ‘BZ-II Image Analysis Application’ build in the system. In the three-dimensional analyses, images were captured, overlaid and reconstruct three-dimension images using ‘Zen 2’ software (Carl Zeiss). In high-resolution analyses, images were captured by LSM800 with Airyscan detector, overlaid and reconstruct three-dimension images using ‘Zen 2’ software. All images displayed in this article are raw images except merged images. In the merged images, only brightness of each channels were adjusted.

## Additional Information

**How to cite this article:** Nagaki, K. *et al*. ePro-ClearSee: a simple immunohistochemical method that does not require sectioning of plant samples. *Sci. Rep.* **7**, 42203; doi: 10.1038/srep42203 (2017).

**Publisher's note:** Springer Nature remains neutral with regard to jurisdictional claims in published maps and institutional affiliations.

## Supplementary Material

Supplementary Information

Supplementary Movie S1

## Figures and Tables

**Table 1:** Clearing times (day) of leaves in ClearSee.

	Whole	Pore	Disk	Slice
*Arabidopsis*	6–7	1–2	6	2–4
Tomato	4	1–2	4	1–2
Tobacco	6	2–3	4–6	2–3
Sunflower	4	1–2	1–2	1–2
Rice	4	1–2	NT	1–2
Wheat	NT	NT	NT	1
Barley	1–2	1	1	1
Maize	NT	NT	2	1
Garlic	NT	NT	NT	1

NT: not tested.

**Table 2:** Immunosignals detectability in ePro-ClearSee.

Species	H3K9me2	H3K4me2	H4Ac	5meC	CENH3	Tubulin
Barley	Detected	Detected	UD	Detected	Detected	Limited area
Wheat	Detected	UD	UD	Detected	Detected	UD
Maize	Detected	Detected	Detected	Detected	UD	Limited area
Rice	Limited area	Detected	UD	Limited area	Detected	NT
Garlic	Detected	NT	NT	NT	UD	NT
*Arabidopsis*	UD	Detected	UD	UD	UD	NT
Tobacco	Detected	Detected	UD	UD	UD	NT
Tomato	Detected	UD	Limited area	UD	NT	UD
Sunflower	Limited area	Limited area	Limited area	UD	Limited area	Limited area

NT: not tested, UD: undetected.

**Table 3:** Comparison of time courses of immunohistochemical methods.

Methods	Fix	Hydrogel polymerization	Enzyme treatment before clearing	Clearing	Enzyme treatment after clearing	Immuno- localization	Total time
PEA-CLARITY	1–2 h on ice	1 day at 37 °C	—	4–6 weeks at 37 °C	5–7 days at 37 °C	2 weeks at 37 °C	7–9 weeks
Sauer *et al*.^16^	60 min at RT	—	—	4 h at 37 °C or RT	30–60 min at 37 °C	6 h at 37 °C	2–3 days
ePro- ClearSee	15 min at RT	—	30–60 min at 37 °C	1–7 days at RT	—	1–2 weeks at 4 °C with 1 h incubation at 37 °C	10 days- 3 weeks
