# Supplementary material for: ePro-ClearSee: a simple immunohistochemical method that does not require sectioning of plant samples
Source: Sci Rep. 2017 Feb 8;7:42203. doi: 10.1038/srep42203 (PMC5297246; doi:10.1038/srep42203)
Supplement: Supplementary Information [file srep42203-s1.pdf]

Title: ePro-ClearSee: a simple immunohistochemical method that does not require sectioning of plant samples

Author names: Kiyotaka Nagaki*, Naoki Yamaji and Minoru Murata

Affiliation and address: Institute of Plant Science and Resources, Okayama University, Kurashiki 710-0046, Japan.

Corresponding author: Kiyotaka Nagaki, E-mail; nagaki@rib.okayama-u.ac.jp, Tel; +81-86-434-1208; Fax: +81-86-434-1208

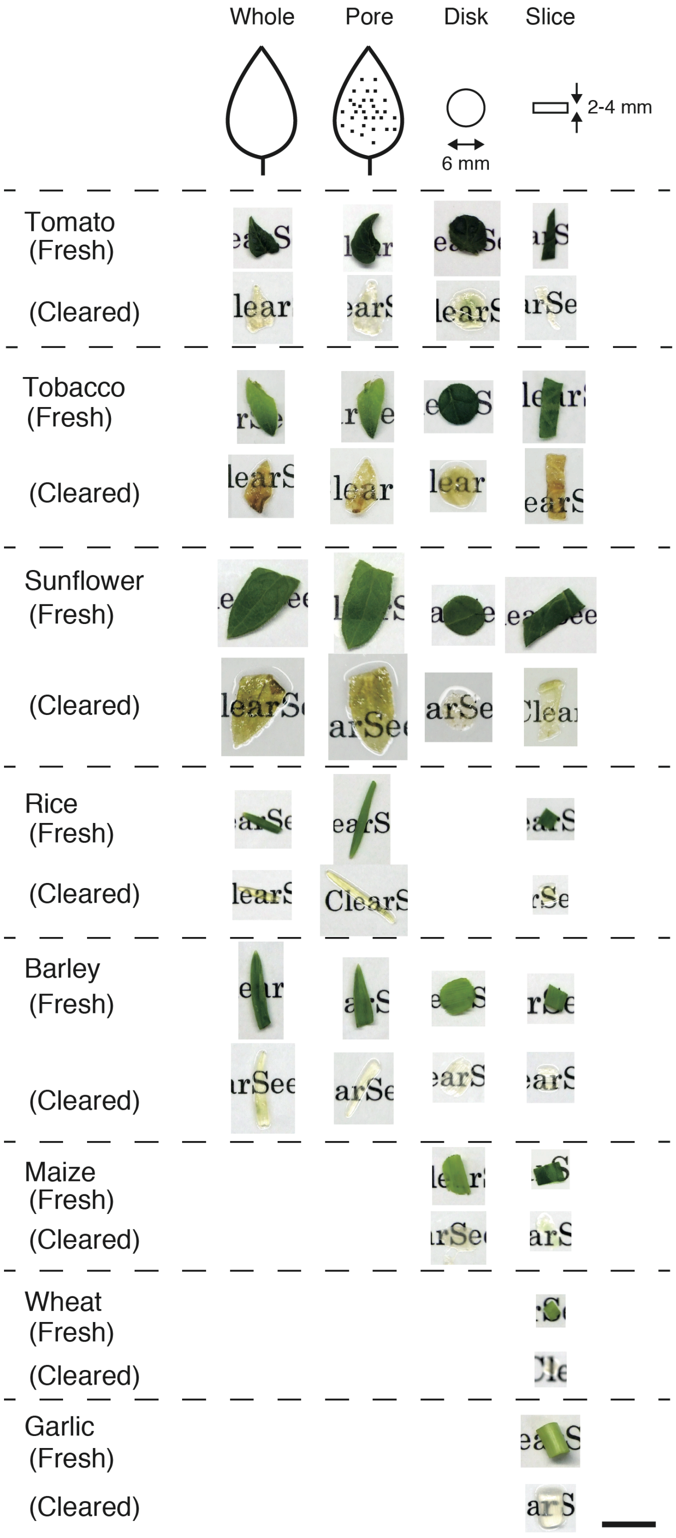

Figure S1. Clearing of leaves by the ePro-ClearSee method. Scale bar, 1 cm.

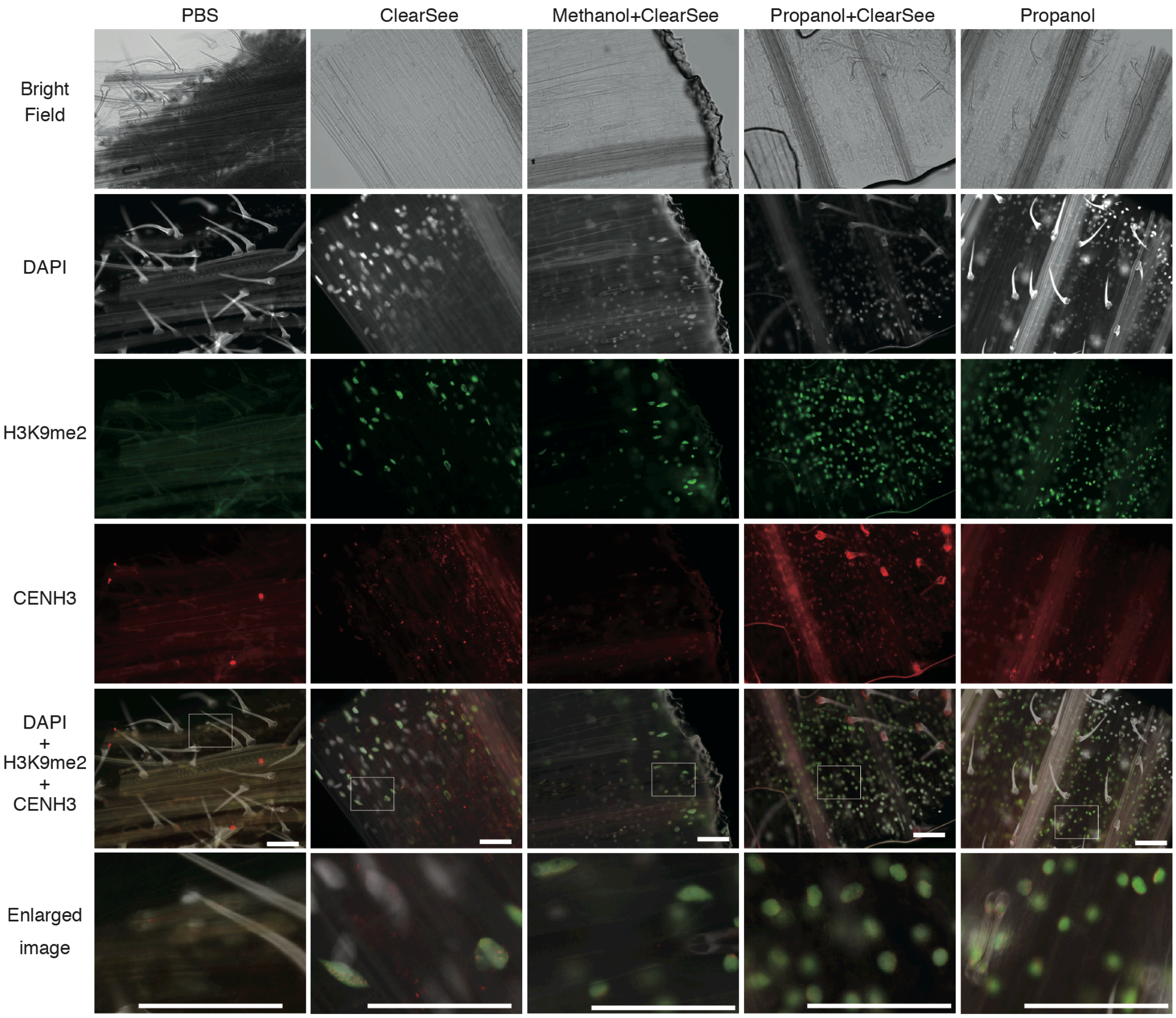

Figure S2. Effects of clearing treatments for wheat leaves. Scale bar, 100 µm.

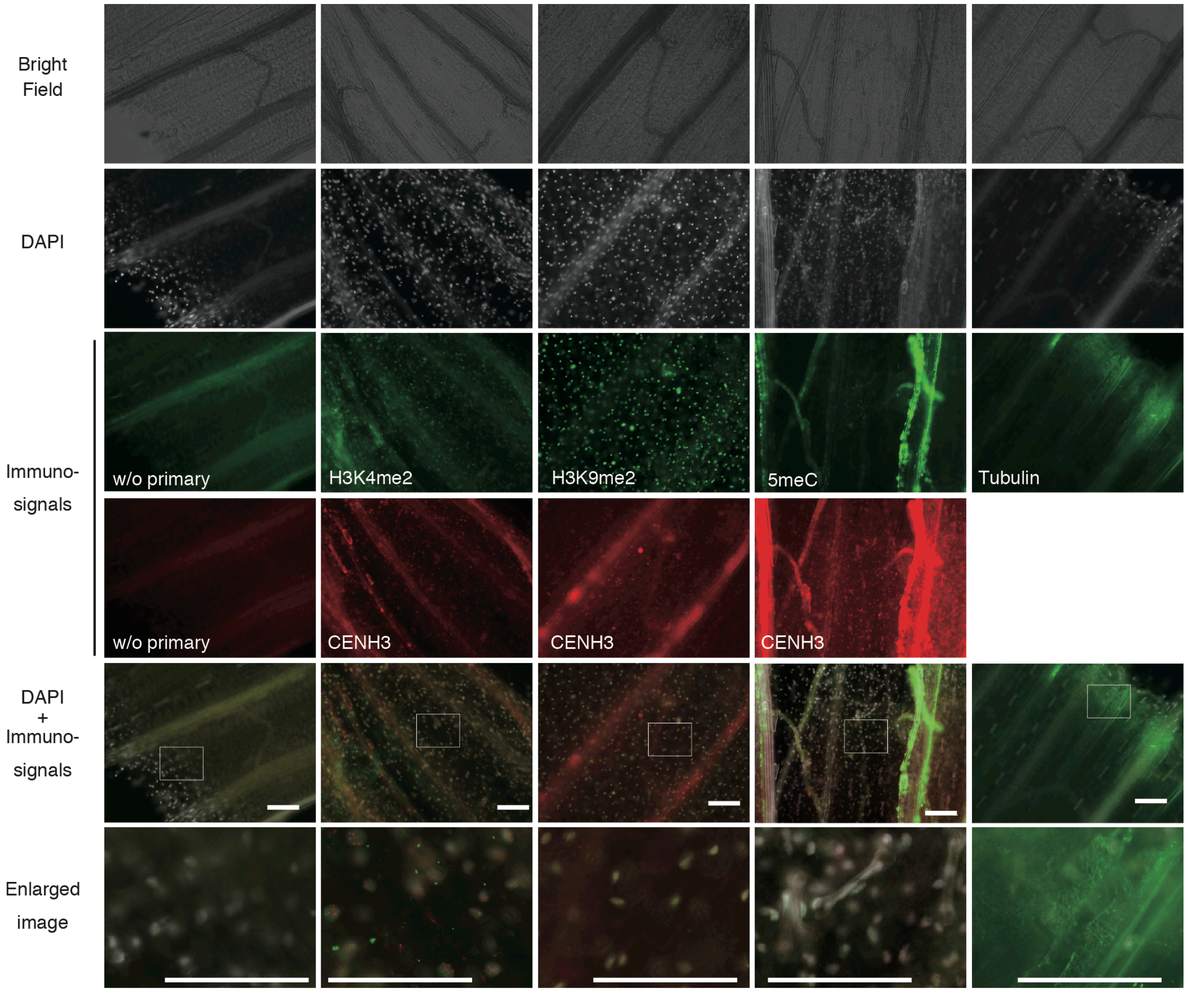

Figure S3. Two-dimensional imaging of ePro-ClearSee treated barley leaves depicting immunosignals of epigenetic modifications and tubulin. Scale bar, 100 μm.

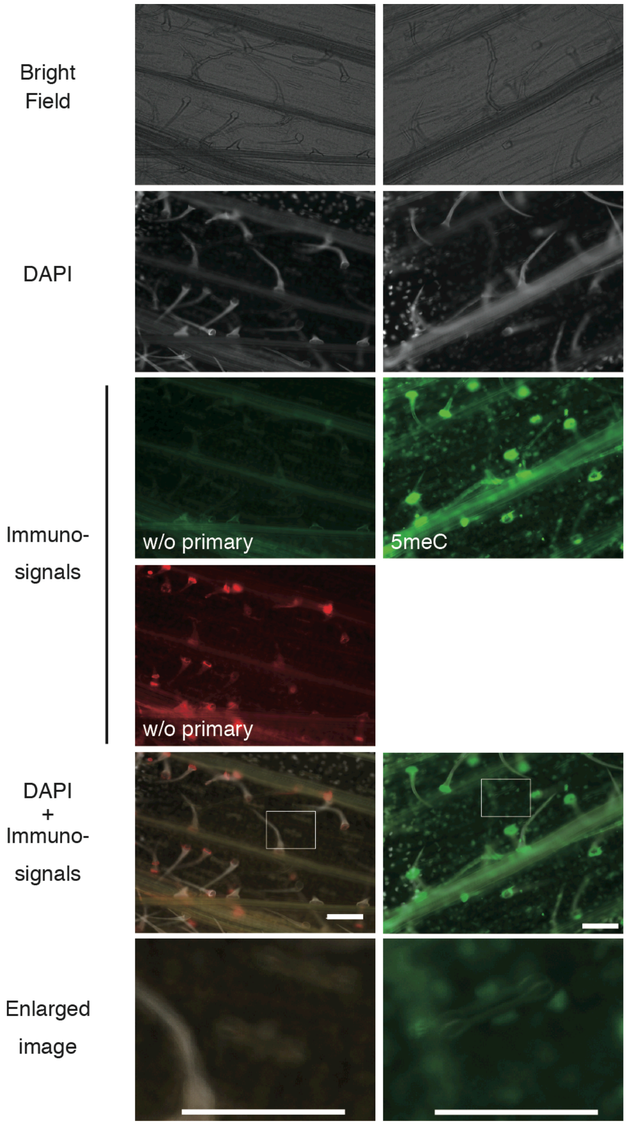

Figure S4. Two-dimensional imaging of ePro-ClearSee treated wheat leaves depicting immunosignals of 5meC. Scale bar, 100 μm.

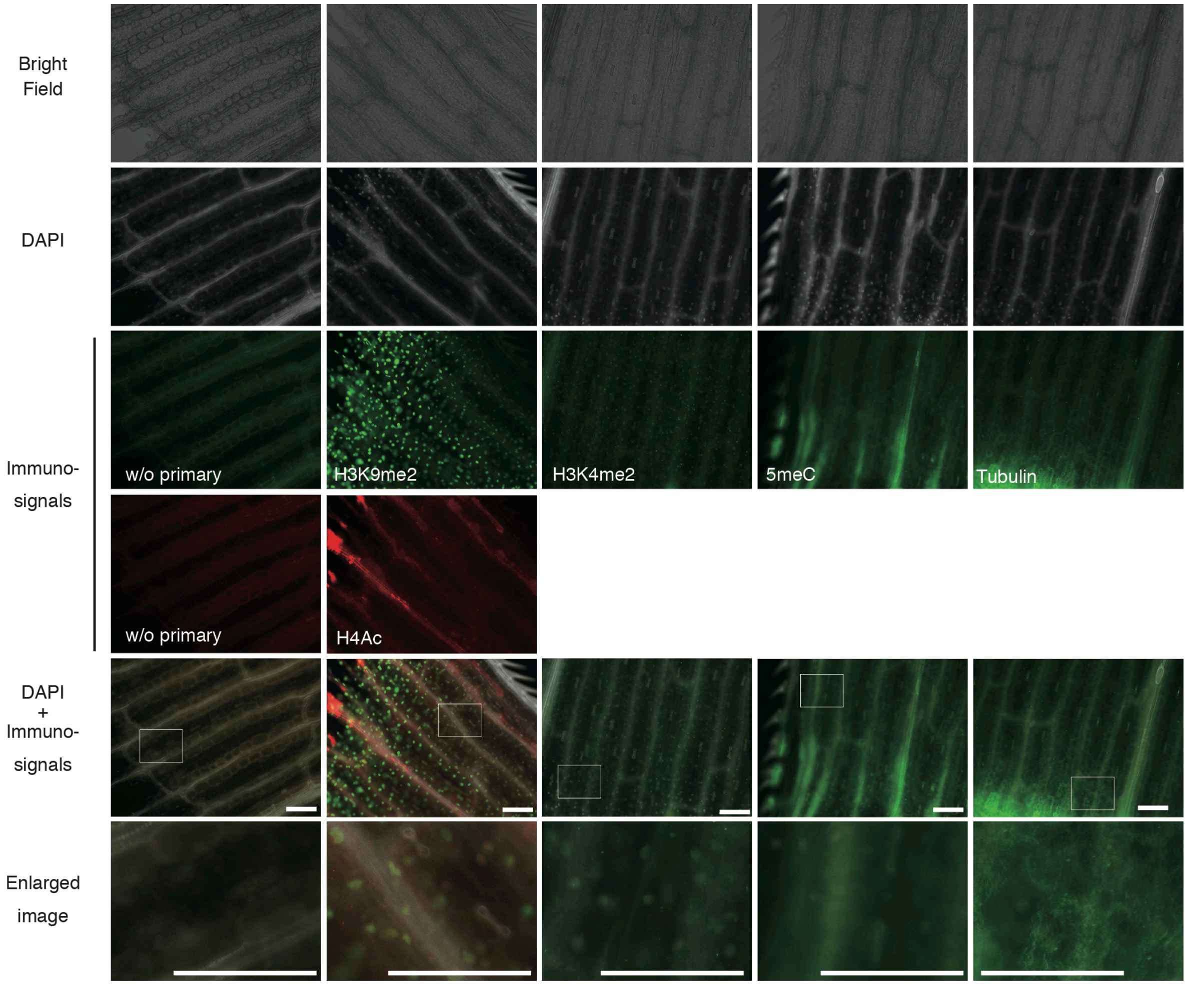

Figure S5. Two-dimensional imaging of ePro-ClearSee treated maize leaves depicting immunosignals of epigenetic modifications and tubulin. Scale bar, 100 μm.

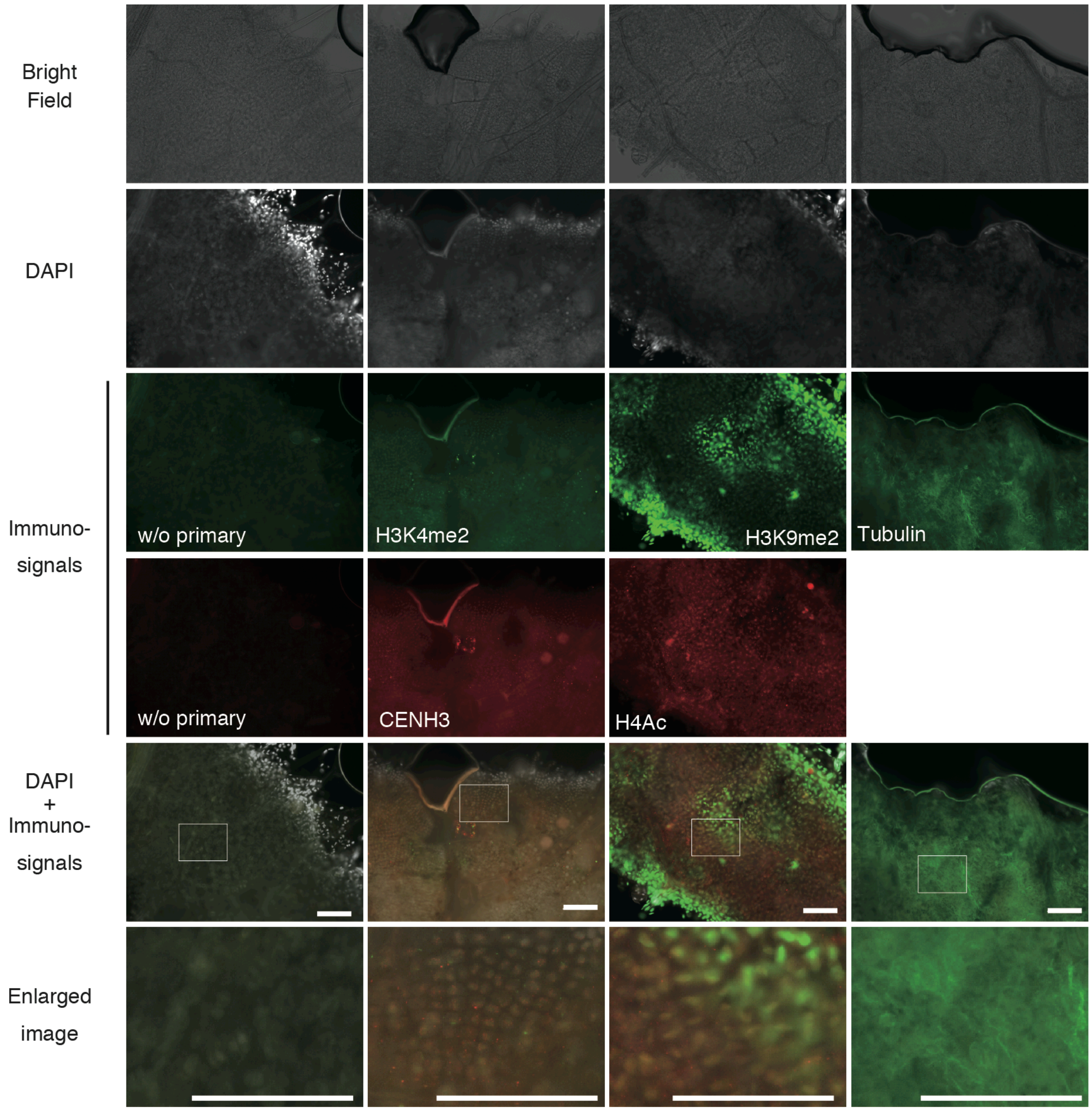

Figure S6. Two-dimensional imaging of ePro-ClearSee treated sunflower leaves depicting immunosignals of epigenetic modifications and tubulin. Scale bar, 100 µm.

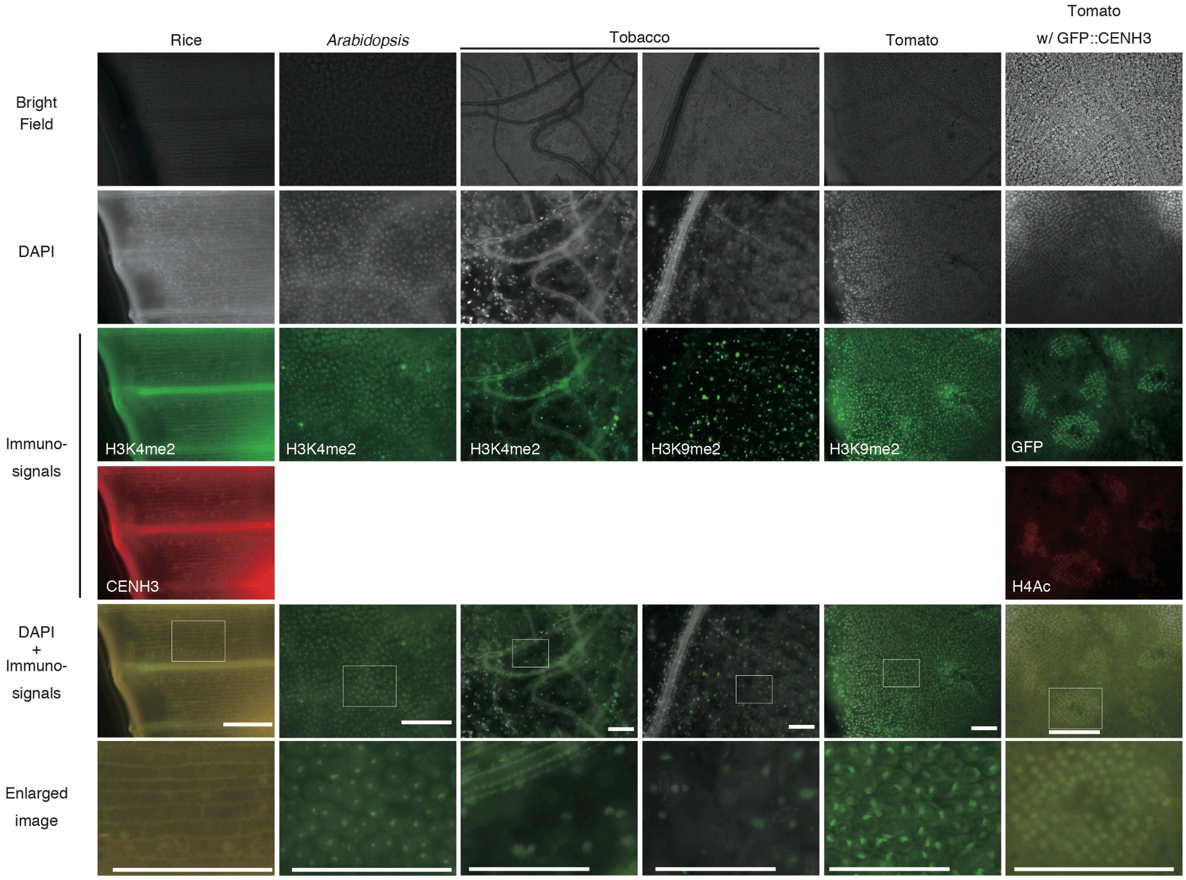

Figure S7. Two-dimensional imaging of ePro-ClearSee treated leaves depicting immunosignals of epigenetic modifications. Scale bar, 100 μm.

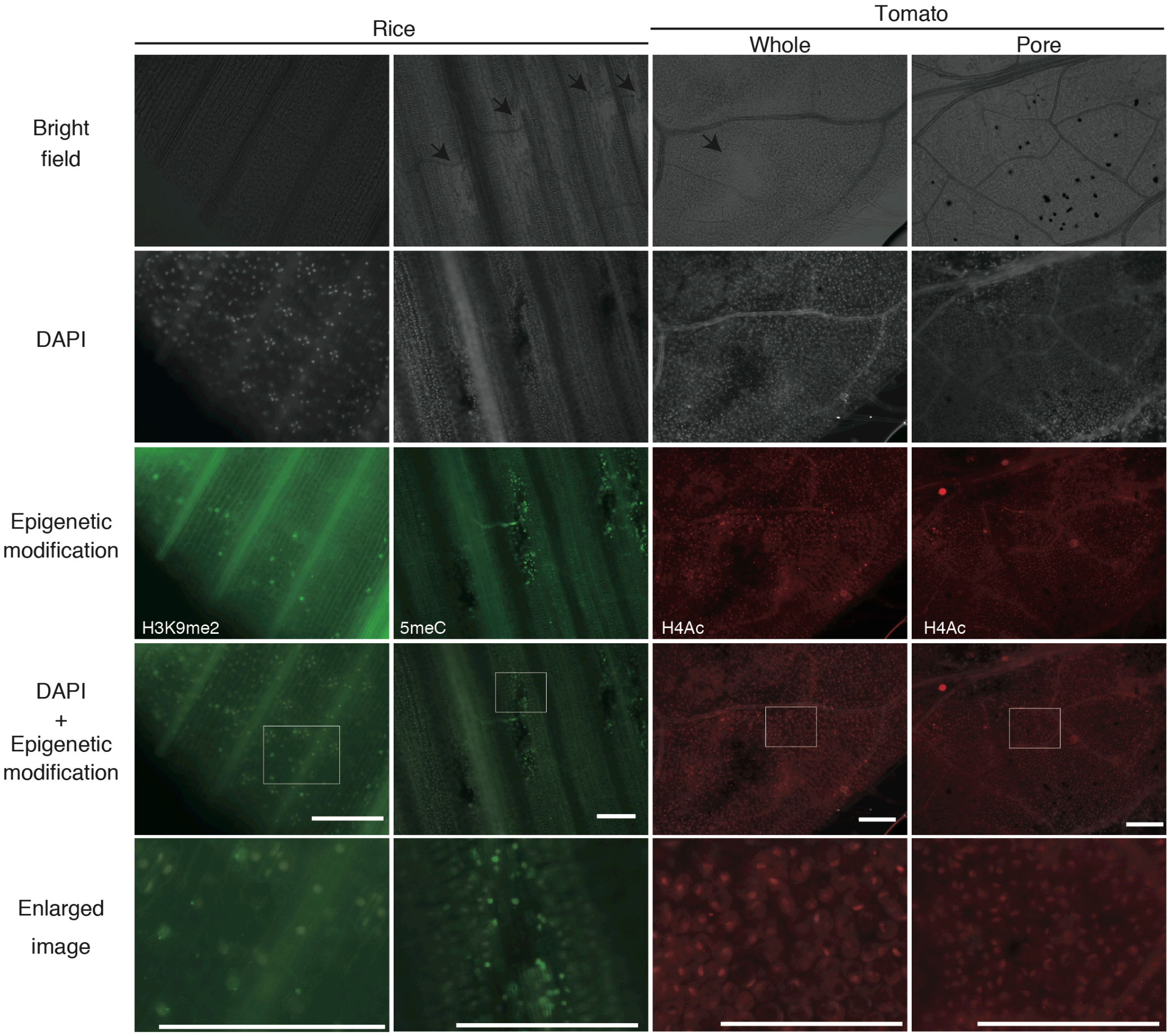

Figure S8. Two-dimensional imaging of ePro-ClearSee treated damaged leaves depicting immunosignals of epigenetic modifications. Scale bar, 100 μm. Arrows in bright field indicate damaged regions. Dots in bright field of tomato 'pore' are pores by a needle.

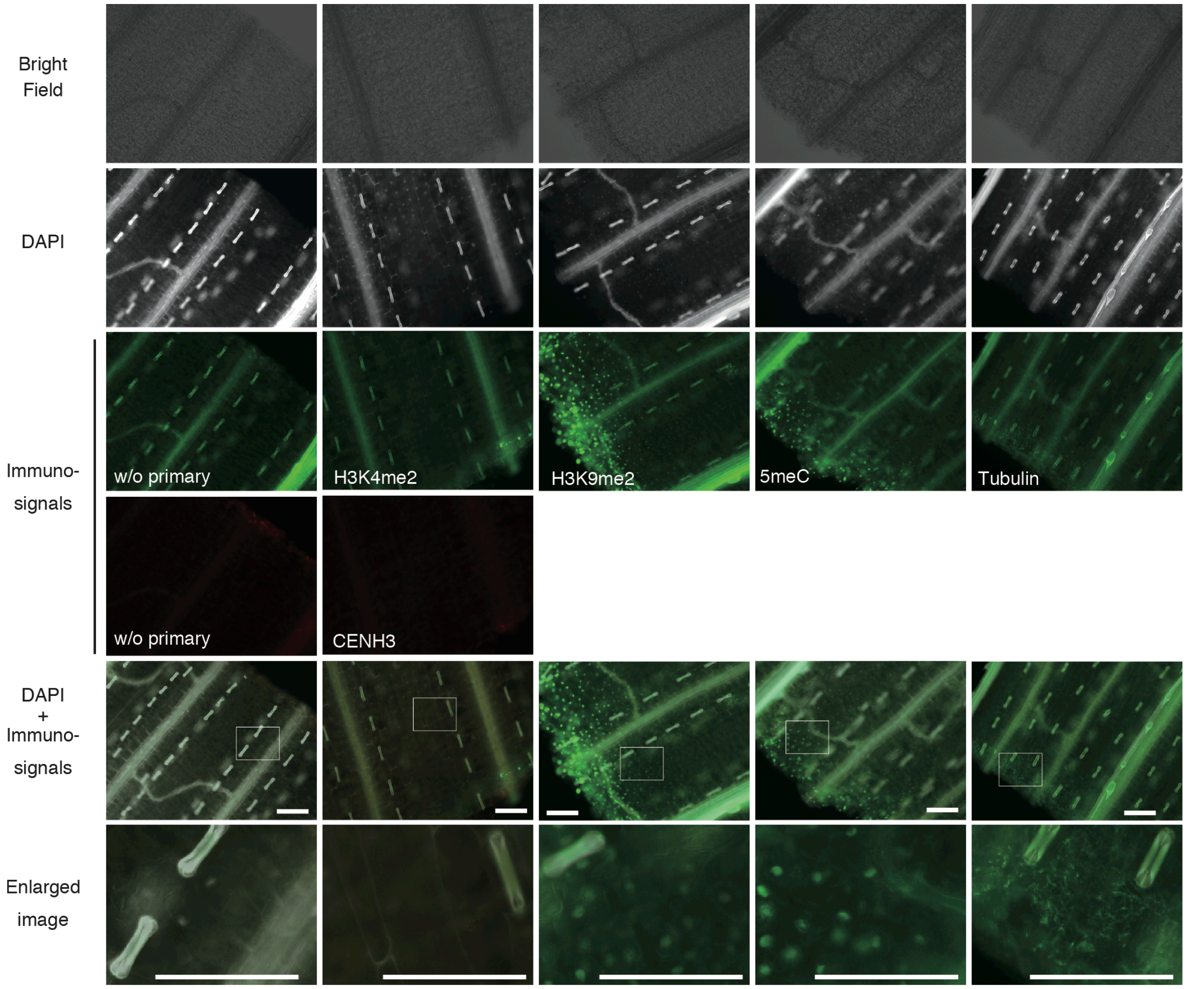

Figure S9. Two-dimensional imaging of barley leaves treated by Sauer's method depicting immunosignals of epigenetic modifications and tubulin. Scale bar, 100 µm.

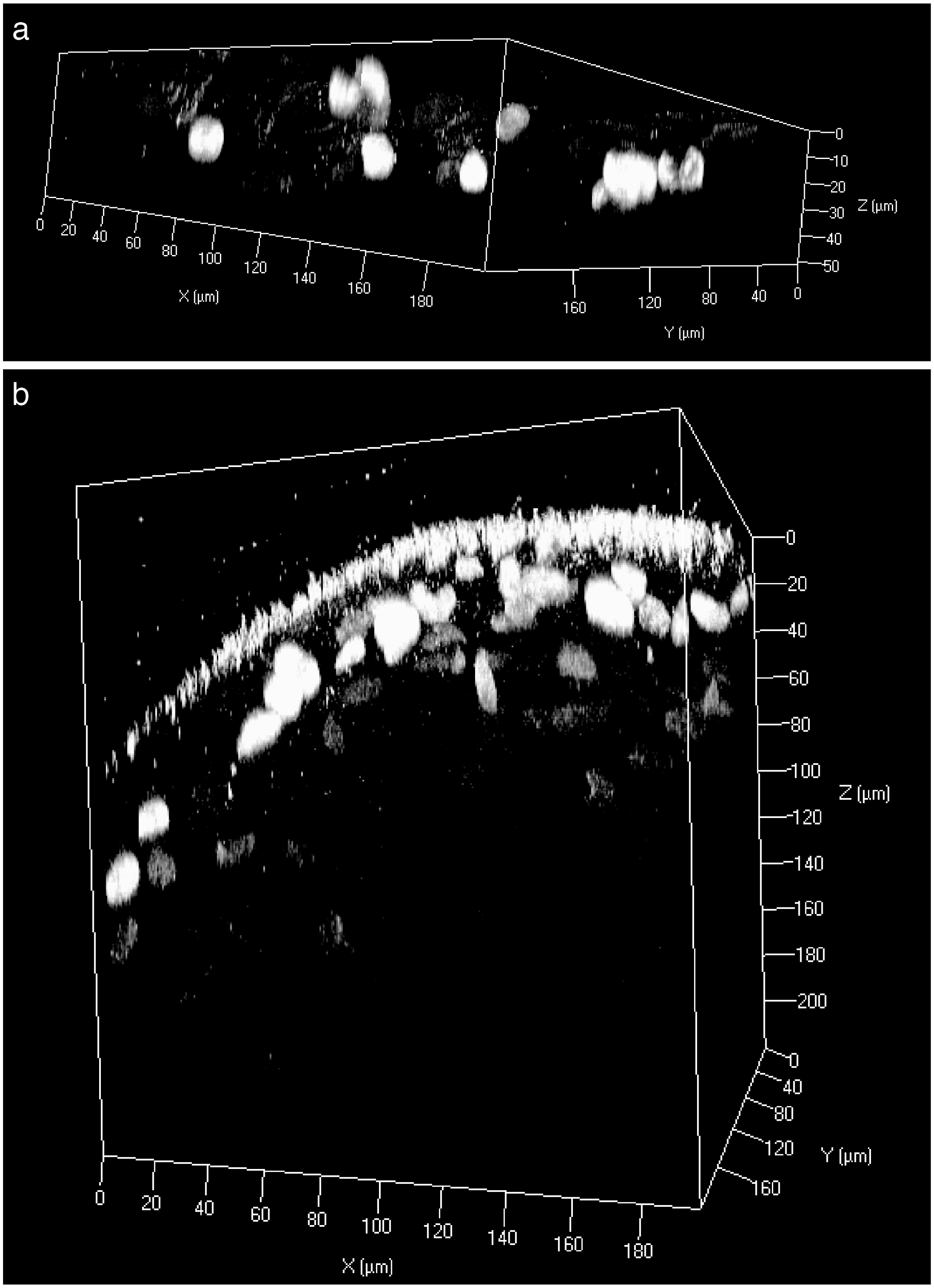

Figure S10. Three-dimensional imaging of an ePro-ClearSee-treated garlic leaf (a) and root (b) depicting immunosignals of di-methylated histone H3 at Lys9.

Movie S1. Optical sections of an ePro-ClearSee treated wheat leaf depicting immunosignals. Immunosignals of di-methylated histone H3 at Lys9 (green) and CENH3 (red) were visualized with DAPI stained nuclei (gray).
